# Venous Thromboembolism (VTE) in May-Thurner Syndrome: Decision-Making Related to Long-Term Anticoagulation

**DOI:** 10.7759/cureus.70674

**Published:** 2024-10-02

**Authors:** Aishwarya Krishnan, Purav Desai

**Affiliations:** 1 Acute Medicine, Calderdale and Huddersfield NHS Foundation Trust, Huddersfield, GBR

**Keywords:** acute medical conditions, cockett’s syndrome, pulmonary embolism (pe), endovascular technique, anticoagulant therapy, ultrasound imaging, deep vein thrombosis (dvt), lower limb edema, • may thurner syndrome (mts), iliac vein compression

## Abstract

May-Thurner syndrome (MTS) involves the compression of the left iliac vein between the right iliac artery anteriorly and the lumbar vertebrae posteriorly. Patients may remain asymptomatic throughout their lives or experience unilateral lower limb swelling and symptoms of deep vein thrombosis (DVT), such as redness and pain in the limb, or features of its complication (pulmonary embolism) such as chest pain or shortness of breath. We present the case of a 34-year-old female exhibiting acute pain and tightness in her left leg, due to DVT of the left common femoral vein, extending up to the pelvic veins, which, on further diagnostic imaging, was found to be due to MTS. The patient was initiated on lifelong anticoagulation to prevent further complications.

The rising incidence of MTS, coupled with frequent delays in its diagnosis, highlights the need to raise awareness among healthcare providers, especially acute medics (who are often the first point of contact for the patient) to expand their diagnostic umbrella of differentials to include MTS as a potential cause of such presentations and to look and think beyond DVT of the lower limb. This is especially important in females presenting with non-specific DVT symptoms, as early suspicion and referral to the respective medical teams including vascular medicine, can improve diagnostic accuracy and provide more management options, thereby improving long-term outcomes.

## Introduction

May-Thurner syndrome (MTS) refers to the occurrence of deep vein thrombosis (DVT), particularly in the left lower limb, due to the compression of the left common iliac vein between the lumbar vertebral body posteriorly and the right common iliac artery anteriorly. While studies by Virchow in 1851 had established that thrombosis of pelvic veins occurred five times more frequently on the left side than on the right side, May and Thurner's study on cadavers in the early 20th century showed the formation of venous spurs in 15-22% of the cases investigated by them - and the term May-Thurner syndrome was eventually coined to refer to this condition [1]. The true incidence of MTS today is unknown but a prevalence of 22-24% was reported in a retrospective analysis of CT scans [2]. Despite the high prevalence of MTS, MTS-related DVT accounts for only 2-3% of all lower extremity DVTs [3,4].

Although MTS can occur in males, 72% of MTS cases in the age group of 20-40 years are reported in females [1,5]. Women present at a younger age compared to men, and gender comparison at presentation has shown a significant difference in the number of men who reported leg swelling and more leg pain compared to women; however, there was no gender-related difference in the reported proportion of patients presenting with DVT. Nevertheless, women were significantly more likely to have a pulmonary embolus on presentation compared to men [6]. 

The obstruction can present at different stages with a variety of symptoms, starting with the compression of the vein, which in itself can cause vague symptoms such as pain and cramps in the lower limb. This is followed by fibrous band formation leading to venous spurs [1,7,8] followed by intimal hypertrophy of the blood vessel in question, which predisposes the patients to clot formation with the Virchow’s triad (stasis, hypercoagulability, and endothelial injury) coming into play as well. The blood flow gets restricted, causing swelling and an increase in the size of the veins along with the pain and sometimes even discoloration of the limb, later leading to ulcers or more acute symptoms such as shortness of breath or chest pain [7], pointing towards the fatal complication of pulmonary embolism.

## Case presentation

A 34-year-old healthy female presented to the emergency department after collapsing without losing consciousness, experiencing increased pain, and feeling tightness in her left lower leg. A systemic review was negative for shortness of breath, chest pain, palpitations, and hemoptysis. A detailed history taking revealed an unremarkable medical history, although her mother had a history of DVT. She had no recent travel history, fever, chills, or trauma to the lower limb that could explain the swelling or pain. She was a non-smoker with no alcohol or substance use or dependency and was not on any regular medications, including contraceptive pills.

Examination of her left lower limb revealed mild swelling without tenderness or warmth. The left leg was approximately 3 cm larger in girth compared to the right one. Both dorsalis pedis and posterior tibialis pulses were palpable. A blood test showed a D-dimer level greater than 9000 ng/mL, while all other results were normal. While awaiting further imaging, she was started on anticoagulation with rivaroxaban 15 mg twice daily for seven days and scheduled for follow-up in the Medical Same Day Emergency Care Unit the next day.

**Figure 1:**
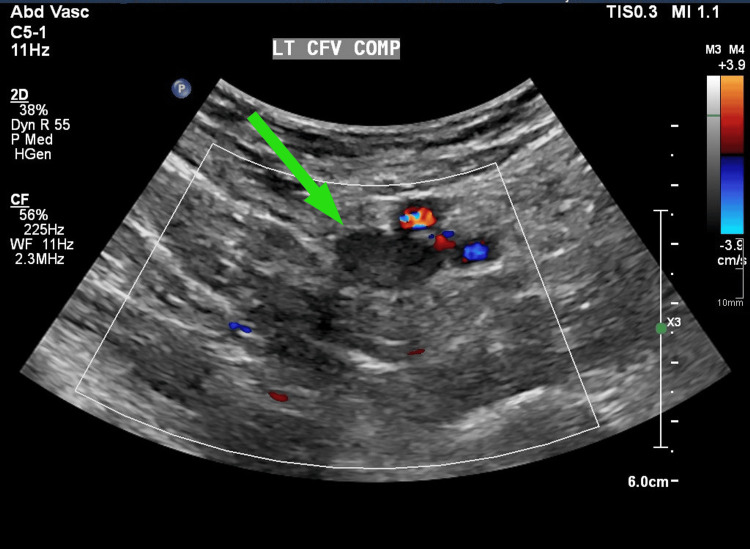
Left common femoral vein compression (CFV COMP) with thrombus within on ultrasound compression venography

**Figure 2:**
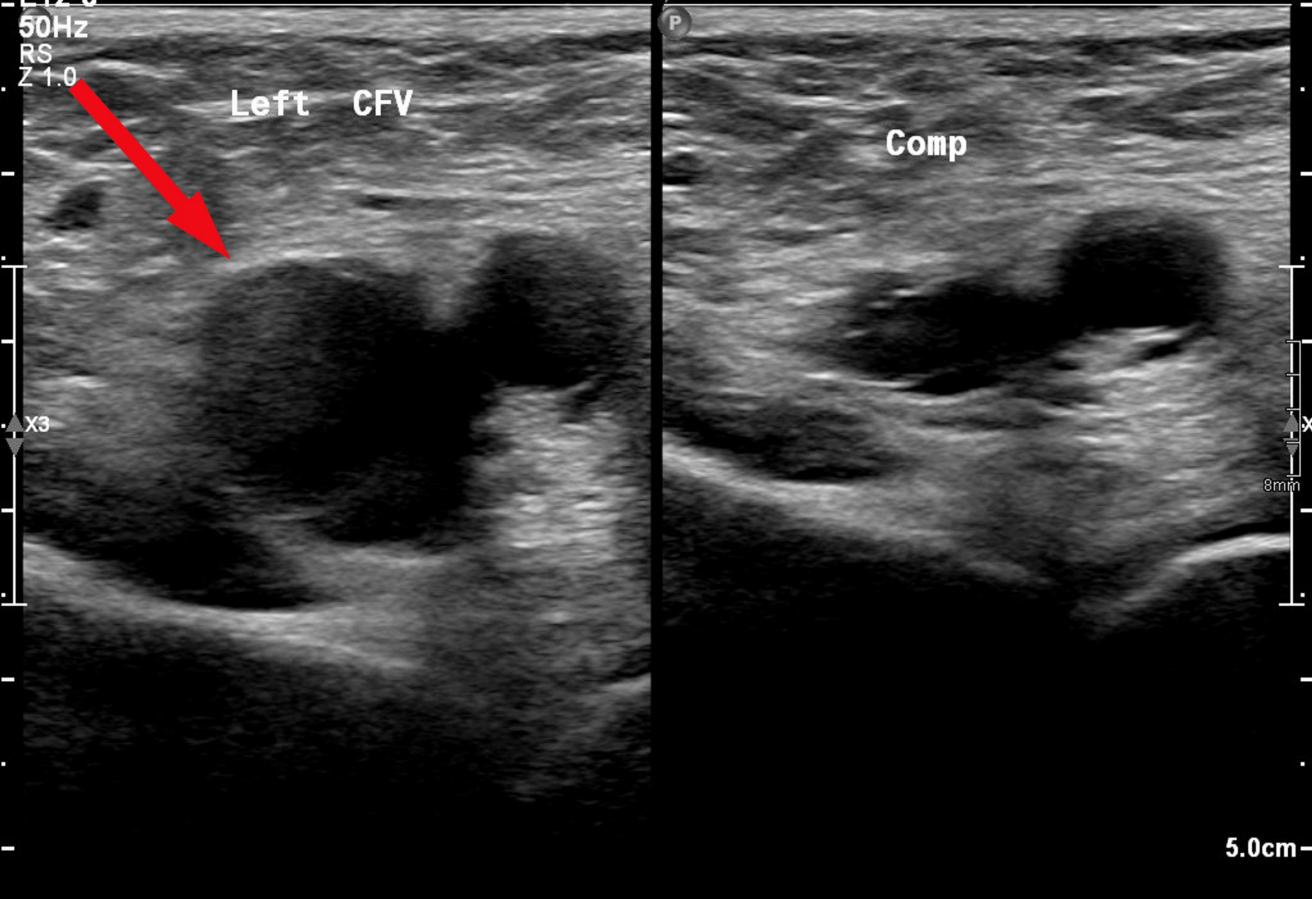
Left common femoral vein (CFV) with thrombus within on ultrasound compression venography before and after compression

Upon review the following day, ultrasound compression venography of her left lower limb showed a thrombus in the left common femoral vein extending into the pelvic veins, and a thrombus in the distal inferior vena cava (IVC) with rouleaux flow in the left superficial femoral vein (Figures 1-2). These findings indicated an unprovoked DVT, and she was continued on anticoagulation, and a follow-up was arranged in the Venous Thromboembolism (VTE) Clinic. She was discharged from the Medical Same Day Emergency Care Unit with safety netting and advised to continue anticoagulation for at least six months.

**Figure 3:**
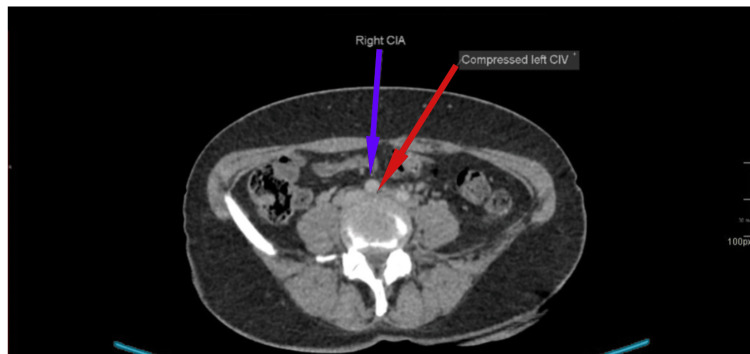
Compressed left common iliac vein (CIV) with right common iliac artery (CIA) in front on CT thorax, abdomen, and pelvis (CT TAP) CT: computed tomography

**Figure 4:**
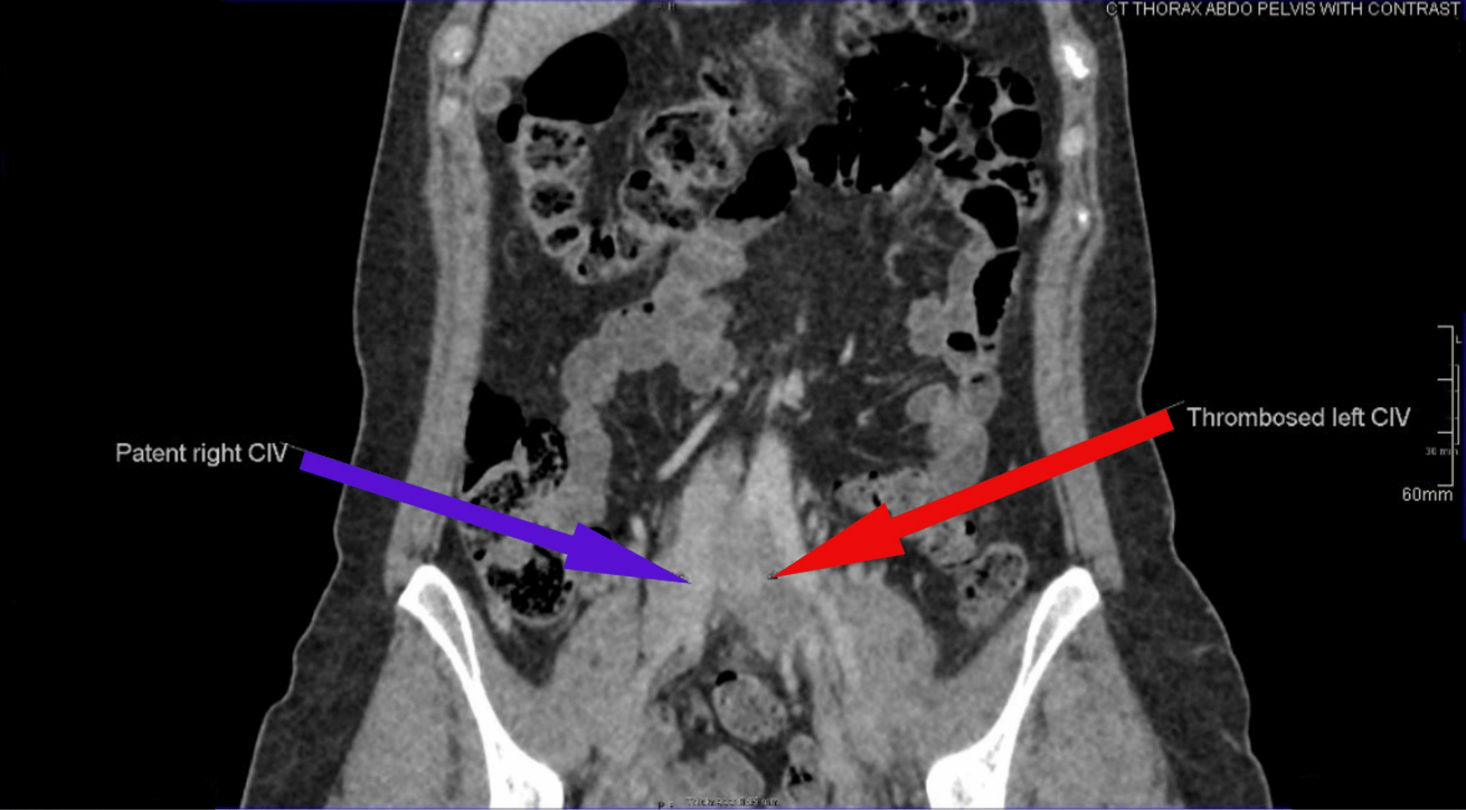
Thrombosed left common iliac vein (CIV) and adjacent patent right CIV seen on CT thorax, abdomen, and pelvis (CT TAP) scan CT: computed tomography

Given the ultrasound findings, a CT scan of the thorax, abdomen, and pelvis (CT TAP) was performed (Figures 3-4). It showed that the left common iliac, external iliac, internal iliac, and common femoral veins were distended with a hypodense filling defect, suggesting an acute thrombus. The left common iliac vein appeared compressed between the right common iliac artery and the L4 vertebral body, indicative of MTS, the likely cause of her DVT.

A vascular opinion was sought based on the CT scan findings, and the patient was referred to the Vascular multi-disciplinary team (MDT). The MDT concluded that the imaging was consistent with MTS. However, because she presented outside the thrombolysis window, and stenting was not indicated, an IVC filter was not deemed necessary. It was decided that she could continue to be managed conservatively with anticoagulation and regular follow-up in the VTE Clinic to monitor her symptoms.

## Discussion

MTS, also known as iliac vein compression syndrome or Cockett syndrome, was first described by Virchow in 1851 as a left-sided predominance of iliofemoral DVT caused by venous stasis disease. However, it was May and Thurner who further investigated this condition comprehensively in 1957 by carrying out an autopsy study on 430 cadavers; they observed compression of the left common iliac vein by the overriding right common iliac artery in 22% of those cadavers [1,4,8]. Retrospective studies analyzing CT scan findings have also shown that a significant proportion of patients had greater than 50% compression of the left iliac vein and that the structure most often compressing the left common iliac vein against the vertebral body was the right common iliac artery (84%) [2].

MTS is a progressive disease and the obstruction to the blood flow in the vein can result either from this anatomical positioning or from chronic intimal hypertrophy induced by the compression itself [9]. The low clinical incidence of MTS-related DVT (2-3%) may be due to missed diagnosis of MTS as the diagnostic workup of DVT is often halted once the underlying common risk factors for DVT are confirmed, such as prolonged bed rest, post-surgical complications, malignancy, or oral contraceptive pills (OCP) [5]. This could also be the reason that MTS is more commonly found in women (who have been on prolonged bed rest, had a recent pregnancy, or have been on OCPs).

A definitive diagnosis of MTS requires a demonstration of the stenotic venous lesion in an appropriate anatomic location [10]. However, many patients remain undiagnosed due to the chronic nature of the condition, which can lead to physiological adaptations such as the formation of collateral circulation to bypass the venous blockage. A delay in diagnosis can pose a life-threatening risk if a clot fragments and travels to the lungs, causing a pulmonary embolism. This also draws our attention to the diagnostic modalities that have improved over the years owing to an increased number of cases being diagnosed and receiving prompt treatment.

Today, there are better diagnostic modalities that help in the early diagnosis of the condition and its management - including ultrasound color Doppler, CT angiography or venography, magnetic resonance venography, and venography with intravascular ultrasound (which remains the gold standard for the diagnosis of MTS). The ultrasound color Doppler is the most commonly used tool; it is a non-invasive modality to diagnose the condition but can often miss a thrombus situated higher up in the limb since it focuses only on the lower extremities. CT scans are considered to be much more sensitive and specific in comparison to color Dopplers. However, they have their own limitations in terms of the ability to control for the volume status of the patient during scanning, which can lead to overdiagnosis of the degree of compression in a dehydrated patient [7,8,11].

A review of the literature revealed a female-to-male ratio of 2:1 for the occurrence and detection of MTS; however, there was no difference in the reported proportion of patients presenting with DVT between men and women [6]. Research suggests that the female predominance observed can be attributed to multiple factors, including the anatomy of the female pelvis that exhibits more of an accentuation of the lumbar lordosis that pushes the lower lumbar vertebrae anteriorly, thereby compressing the left common iliac vein against the right common iliac artery; recent pregnancy; or the use of OCPs [8].

A recent study by Choi et al. in 2015 involving a cohort of 201 patients with DVT showed that 137 of them had MTS that required endovascular treatment [12]. Conservative management remains the primary treatment approach and involves using anticoagulants to prevent recurrent clot formation and subsequent pulmonary embolism. These anticoagulants may include low-molecular-weight heparin, unfractionated heparin, newer factor Xa inhibitors, or direct oral anticoagulants (DOACs). However, anticoagulation alone might be insufficient in some cases and various surgical treatments, including minimally invasive endovascular techniques, which have been developed over the years, might need to be considered. The surgical options also include vein-patch angioplasty with the removal of intraluminal bands, division and relocation of the right common iliac artery behind the left common iliac vein or IVC, and contralateral saphenous vein graft bypass to the ipsilateral common femoral vein with the creation of a temporary arteriovenous fistula (Palma crossover) [9].

The most common treatment combination found in the literature is catheter-directed thrombolysis, followed by iliac vein stenting and subsequent anticoagulation. The study by Comerota in 2002-03 specifically looked at the quality-of-life improvement when using thrombolytic therapy for iliofemoral DVT and showed that following treatment, patients who received catheter-directed thrombolysis reported better overall physical functioning, less stigma, less health distress, and fewer postthrombotic symptoms compared to those treated with anticoagulation alone. Successful lysis was also seen to correlate with improvement in health-related quality of life [13]. As with any procedure, surgical treatments for MTS do pose potential complications, including acute renal failure, vascular injury, bleeding, and device complications such as catheter tip detachment. In some cases, post-thrombotic syndrome (characterized by diverse signs and symptoms of venous insufficiency, such as pain, swelling, paraesthesia, skin hyperpigmentation, and venous ulceration), a frequent complication of DVT, can also be seen despite a conservative approach with optimal anticoagulation [14]. 

Surgical thrombectomy is an option for patients with DVT that is unresponsive to thrombolytic therapy or for whom thrombolysis is contraindicated; however, it is rarely used and a retrievable IVC filter may also be considered for patients with a preexisting pulmonary embolism. Thrombolysis also carries a risk of hemorrhagic complications, including stroke [7]. But even though compression of the left iliac vein is likely to lead to a higher prevalence of left-sided DVT, a recently published expert opinion review supports has indicated that it may be protective against pulmonary embolism, acting like an "anatomical filter" for emboli [15].

## Conclusions

Given the increased incidence of MTS and the vast variety of diagnostic modalities available today, it should be considered in young women presenting with symptoms of DVT. A high clinical suspicion should prompt a discussion about further investigations to exclude underlying hypercoagulable conditions including the presence of protein C, protein S, antithrombin III deficiency, factor V Leiden mutation, prothrombin gene mutation, antiphospholipid antibody syndrome, and hyper-homocysteinemia, all of which could contribute to the formation of clots and recurrence of DVT. Early intervention can also help prevent long-term complications such as pulmonary embolism and phlegmasia cerulea dolens, which increase morbidity and mortality. This report highlights a relatively rare but significant condition, MTS, as the underlying cause of unprovoked DVT in a young female. It lays stress on the importance of a comprehensive diagnostic workup and the role of imaging in guiding management. Addressing the potential areas for improvement will enhance the educational value and practical implications for clinicians. Overall, the report contributes to the understanding of DVT management and the clinical implications of specific venous anatomy.
